# Role of Epstein–Barr Virus C Promoter Deletion in Diffuse Large B Cell Lymphoma

**DOI:** 10.3390/cancers13030561

**Published:** 2021-02-01

**Authors:** Seiyo Mabuchi, Fumiya Hijioka, Takahiro Watanabe, Yusuke Yanagi, Yusuke Okuno, H. M. Abdullah Al Masud, Yoshitaka Sato, Takayuki Murata, Hiroshi Kimura

**Affiliations:** 1 Department of Virology, Nagoya University Graduate School of Medicine, Nagoya 466-8550, Japan; mabuchi.seiyo@med.nagoya-u.ac.jp (S.M.); fhijioka@mail.biken.or.jp (F.H.); t.nabe.watanabe@med.nagoya-u.ac.jp (T.W.); yusuke.yanagi@med.nagoya-u.ac.jp (Y.Y.); masud.mbio@cu.ac.bd (H.M.A.A.M.); yssato@med.nagoya-u.ac.jp (Y.S.); 2 Department of Pathology and Laboratory Medicine, Nagoya University Graduate School of Medicine, Nagoya 466-8550, Japan; 3 Medical Genomics Center, Nagoya University Hospital, Nagoya 466-8550, Japan; yusukeo@med.nagoya-u.ac.jp; 4 Department of Microbiology, Faculty of Biological Sciences, University of Chittagong, Chattogram 4331, Bangladesh; 5 Department of Virology and Parasitology, Fujita Health University School of Medicine, Toyoake 470-1192, Japan

**Keywords:** EBV, DLBCL, C promoter, LMP2A, BHRF1, growth transformation

## Abstract

**Simple Summary:**

The C promoter of Epstein–Barr virus is assumed to be important for B cell growth and transformation. However, we present evidence that promoter activity is not only unneeded for transformation but also that absence of the promoter increased the transformation activity of the virus. We found that the C promoter was lost in some Epstein–Barr virus-associated lymphoma specimens. Therefore, deletion of the promoter could partially account for the tumorigenesis of Epstein–Barr virus-associated lymphomas.

**Abstract:**

The Epstein–Barr virus (EBV) is the cause of several malignancies, including diffuse large B cell lymphoma (DLBCL). We recently found that EBV genomes in EBV-positive cancer specimens have various deletions (Okuno et al. Nat Microbiol. 2019). Here, we focus on the deletion of C promoter (Cp), which transcribes EBV nuclear antigen (EBNA) genes in type III latency. The Cp deletion found in a DLBCL patient (332 bp) was introduced into EBV-BAC of the B95-8 strain. Interestingly, the dCp virus transformed B cells more efficiently than WT and revertant strains. Deletion of Cp also promoted tumor formation and severe pathogenicity in a mouse xenograft model. RNA sequencing and qRT–PCR analyses revealed that Cp transcription was undetectable in the dCp cells. Instead, transcription from the W promoter (Wp), an alternative promoter for EBNA, was activated in the dCp mutant. We also found that the expression of latent membrane protein 2A (LMP2A) was somehow induced in the dCp mutant. Double knockout of Cp and LMP2A indicated that LMP2A is crucial for B cell transformation, but the increased transformation induced by Cp deletion cannot be explained by LMP2A alone. We also tested the effect of an anti-apoptotic viral BCL2 homolog, BHRF1, because its expression was reportedly induced more efficiently by that of Wp. However, increased growth transformation via Cp deletion was not due to the BHRF1 gene. Taken together, the results indicated that deletion of a specific region in Cp increased in vitro transformation and the rate of progression of EBV-positive lymphoproliferative disorders in vivo. Our data suggest that genomic alteration not only of the host but also the virus promotes EBV-positive tumor generation and expansion, although the molecular mechanism underlying this phenomenon is still unclear. However, LMP2A and BHRF1 are not involved.

## 1. Introduction

The Epstein–Barr virus (EBV) is a member of the human gamma-herpesvirus family, which was first discovered in endemic Burkitt lymphoma (BL) cells. EBV contains a large double-stranded DNA genome (~170 kbps) encoding over 80 genes and 40 mature microRNAs (miRNAs) and noncoding RNAs. It is a ubiquitous virus present in over 90% of the worldwide population and primarily infects B cells via the saliva during childhood. Most cases are asymptomatic, but it occasionally manifests as infectious mononucleosis.

After the primary infection, the virus suppresses the expression of most viral genes, hiding predominantly in memory B cells without virus production nor presenting obvious symptoms (latent infection). Latent EBV expresses its genes in different patterns according to latency programs, categorized as type 0–III. For example, EBV can infect peripheral blood B cells in vitro, resulting in transformation into continuously proliferating lymphoblastoid cell lines (LCLs). The LCLs take type III latency and express all latent EBV genes. Regardless, after some time, EBV can reactivate from latency and enter its lytic cycle, resulting in the production of progeny virus.

A small but a significant number of EBV-positive people develop lymphomas, including BL, Hodgkin lymphoma, diffuse large B cell lymphoma (DLBCL), chronic active EBV infection (CAEBV), extranodal natural killer (NK)/T cell lymphoma, nasal type (ENKL), nasopharyngeal carcinoma, and gastric cancer. The reason why these cancers affect some EBV-positive patients is still incompletely understood [1,2].

EBV is associated with tumor growth and proliferation through multiple mechanisms. EBV contains oncogenes such as latent membrane protein 1 (LMP1) and LMP2A. These genes mimic CD40 and BCR, respectively, and activate downstream signaling pathways [3,4]. LMP1 and LMP2A have been shown to mediate Reed-Sternberg cell survival and proliferation in Hodgkin lymphoma. Another EBV gene, BNRF1, induces genomic alterations and chromosomal instability in the host genome through amplifying centrosome activity, resulting in genomic mutations [5]. EBV nuclear antigen 2 (EBNA2) binds to super-enhancer regions and modifies gene transcription, such as by upregulating the MYC oncogene [6]. LMP1 and EBNA2 allow the virus to escape immune detection through upregulation of PD-L1 [7,8].

**Figure 1:**
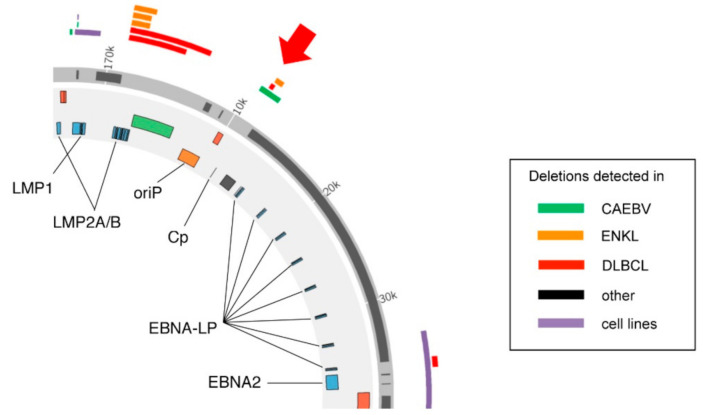
Deletion of the Epstein–Barr virus (EBV) genome in EBV-positive cancer specimens. Map of the deletions in C promoter (Cp) found in our previous study (Okuno et al. Nature Microbiology, 2019). These deletions were present in chronic active EBV infection (CAEBV), EBV-positive diffuse large B-cell lymphoma (DLBCL) and extranodal natural killer (NK)/T-cell lymphoma, nasal type (ENKL) patients. Modified from (Murata et al. Reviews in Medical Virology, 2020).

Until recently, it was believed that EBV in cancers always had a complete set of genes in the full-length viral genome without any deletions, except a few rare cases, such as Wp-restricted BL [9]. However, we recently found that the viral genome in EBV-associated lymphomas and lymphoproliferative disorders bears several types of deletion [10,11]. Especially, we found deletion of the EBV C promoter (Cp) in cases of DLBCL, ENKL and CAEBV (Figure 1). Cp is an EBV promoter that mediates the transcription of EBNAs in type III latency cells, such as LCLs. Upon EBV infection of B cells, the W promoter (Wp) is activated and produces EBNA proteins, including EBNA2. As the expression of EBNA2 protein increases, it activates Cp and other downstream promoters and suppresses Wp [12,13]. Hence, the Wp is activated for expression of EBNAs immediately after infection, but then within a week or two, the Cp takes over the expression of EBNAs, although the physiological significance of this promoter switching is not understood yet.

We are interested in the role of Cp deletion in the generation of EBV-associated tumors. To elucidate the mechanism, we introduced a Cp deletion into an EBV-bacterial artificial chromosome (BAC) and compared the phenotype between Cp-deficient (dCp) virus and the wild-type (WT) virus. Interestingly, disruption of Cp resulted in a 10-fold higher rate of B cell growth transformation. We examined the role of LMP2A and BHRF1 in the increased immortalization seen in the dCp virus infection but found no connection. Therefore, deletion of the Cp in a DLBCL was confirmed to be beneficial for tumorigenesis, but the molecular mechanism remains unknown.

## 2. Results

### 2.1. Construction of Cp-Deficient EBV-BAC Strain

Previously, we demonstrated that EBVs latently infected in a portion of human lymphoma specimens have intragenic deletions [10]. These deletions were most frequently found in the BamHI-A rightward transcript (BART) region and essential lytic genes. Loss of the BART region increased lytic gene expression and tumor formation in a mouse xenograft model [14]. Disruption of BALF5 showed a similar phenotype [10]. Several of the deletions found in CAEBV, ENKL, and DLBCL tissue biopsies affected Cp (Figure 1). The discovery of these deletions in clinical specimens was surprising because it seemed to contradict previous reports that the loss of Cp expression resulted in lower B cell growth transformation activity [15,16]. As CAEBV and ENKL affect both T and NK cells, we decided to focus on the Cp deletion seen in the DLBCL specimen.

**Figure 2:**
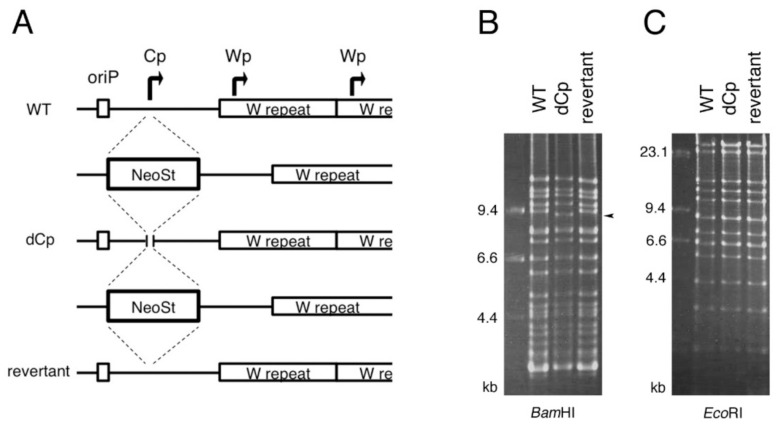
Construction of the C promoter deletion (dCp) and revertant (rev) EBV mutant constructs using the EBV-bacterial artificial chromosome (BAC) system. (**A**) Schematic diagram of the EBV-BAC recombination. The Neo/St cassette was inserted first in the Cp. Neo/St cassette was replaced with a Cp sequence with deletion from 11,145 to 11,476 (332 bp) of KC207813.1. The Neo/St cassette was reinserted again and then replaced with the wild-type (WT) Cp sequence to construct the rev virus. (**B**,**C**) Recombinant EBV-BAC DNA was digested by BamHI and EcoRI, and the products were examined by agarose gel electrophoresis. Note that the digested product near 9.4 kbp was shortened in the dCp virus (indicated by arrowhead), reflecting the deletion.

To investigate the effect of Cp deletion on the EBV life cycle, we constructed a Cp-deficient recombinant EBV using the EBV-BAC system (Figure 2). We first inserted the neomycin-resistant/streptomycin-sensitive (Neo/St) gene cassette into WT EBV-BAC DNA (B95-8 strain) using homologous recombination. Then the Neo/St cassette was replaced with a Cp sequence including a deletion, i.e., dCp. A revertant (rev) strain was prepared by inserting and then removing the Neo/St cassette (Figure 2A). To confirm the integrity of the viral genome, recombinant EBV-BAC DNA was digested with BamHI or EcoRI, and the digested products were examined by electrophoresis (Figure 2B,C). The band patterns of the three strains (WT, dCp, and rev) were identical aside from a digested product near 9.4 kbp that was shortened in the dCp by digestion with BamHI (arrowhead), reflecting that the intended Cp deletion was achieved. These results indicate that the recombinant viral genomes were intact and did not have obvious unexpected deletions or insertions.

These EBV-BAC DNAs were transfected to HEK293 cells by lipofection. Hygromycin resistant green fluorescent protein (GFP)-positive colonies were collected, in which the recombinant EBV genome is latently maintained. Cells were transfected with a BZLF1-expressing vector to activate the lytic cycle of EBV and induce progeny virus production. We confirmed that deletion of the Cp had little or no effect on the production of progeny EBV production, at least in HEK293 cells (not shown). The viral titers were determined by infecting Akata(−) cells and used for the transformation assays.

### 2.2. Cp Deletion Increases B Cell Transformation Efficiency

**Figure 3:**
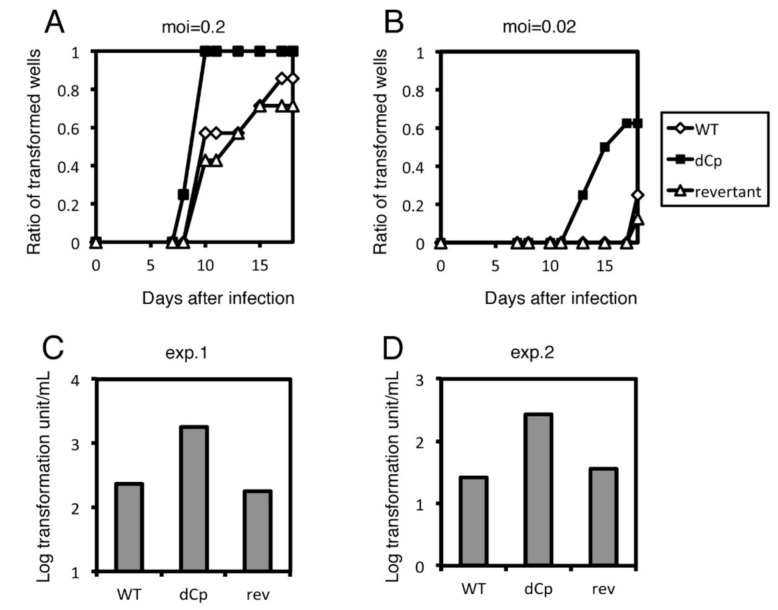
Transformation of WT, dCp and rev viruses. Recombinant viruses (WT, dCp and rev) were produced from HEK293 cells. After titer normalization of the viruses, 10-fold serial dilutions were prepared and used to separately infect peripheral blood mononuclear cells (PBMCs). For each dilution, 8 wells were prepared. (**A**,**B**) Ratio of transformed wells with a multiplicity of infection (MOI) of 0.2 (**A**) and 0.02 (**B**) over time. (**C**,**D**) Transformation efficiency at 3 weeks post-infection. Two independent experiments were carried out using PBMCs from two healthy donors (exp. 1 and exp. 2).

To examine the role of Cp deletion in B cell growth transformation, we infected peripheral blood mononuclear cells (PBMCs) from healthy donors with the three viral strains (WT, dCp and rev) described above (Figure 3). At a multiplicity of infection (MOI) of 0.2, WT and rev strains caused obvious cell clump formation in about 50% of wells, compared to 100% of the wells treated with the dCp virus at day 10 (Figure 3A). A similar trend was observed in the samples infected at a lower MOI (0.02) (Figure 3B). Transformation efficiency was calculated after 3 weeks of incubation. The log transformation units per milliliter were 2.38, 3.25 and 2.25 in WT, dCp, and rev, respectively (Figure 3C). Reproducibility was confirmed by using PBMCs from a second donor, where the WT, dCp, and rev EBVs exhibited 1.43, 2.43 and 1.55 log transformation units per milliliter, respectively (Figure 3D). Therefore, the dCp EBV caused increased B cell transformation compared to controls.

### 2.3. dCp Virus Increased Pathogenicity in an EBV-Associated LPD Mouse Model

**Figure 4:**
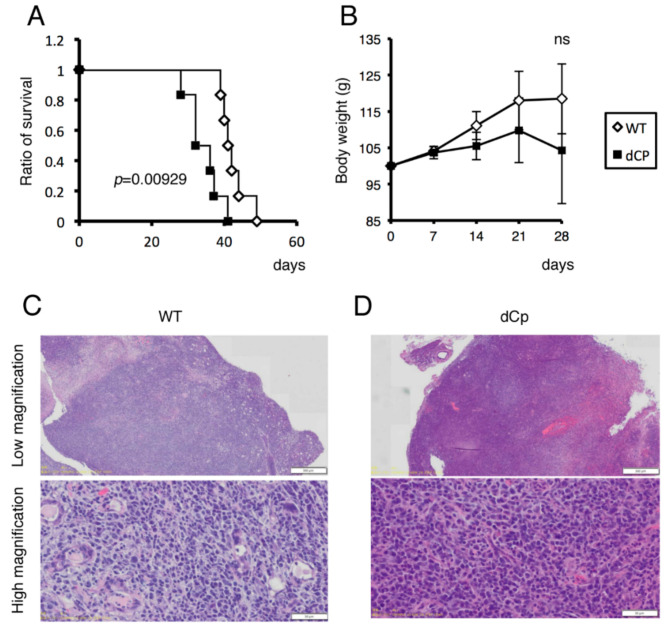
Survival of xenograft mice with EBV-associated lymphoproliferative disorder. First, 1.0 × 10^7^ human cord mononuclear blood cells were each infected with 1.0 × 10^4^ viruses (WT, dCp) and immediately transplanted intraperitoneally into NOG mice (*n* = 6). (**A**,**B**) Survival ratio (**A**) and body weight (**B**) of the mice were plotted. (**C**,**D**) Microscopic images of H-E staining of tumors in the pancreas of the mice infected with WT (**C**) or dCp (**D**) virus.

We next examined the influence of Cp deletion in vivo using a mouse xenograft model. Human cord blood mononuclear cells were infected with WT or dCp virus and immediately injected intraperitoneally into NOG mice. Mice receiving dCp virus-infected cells showed a higher mortality rate compared to controls (Figure 4A) and more severe weight loss (Figure 4B), although the body weight loss was not statistically significant. Tumors formed in the pancreas of the mice were then subjected to hematoxylin–eosin (H-E) staining. Infection of either WT or dCp resulted in tumor formation, but the mice with the dCp virus exhibited more extensive infiltration of tumor cells (Figure 4C,D). These results imply that Cp deletion also plays an important role in EBV-associated tumor progression in vivo. We here analyzed tumors in the pancreas because they were macroscopically detected easily than those in other organs, such as the spleen and liver, but there were tumors in other organs, too.

### 2.4. LCLs with the dCp EBV Show Increased Expression of LMP2A and LMP2B

**Figure 5:**
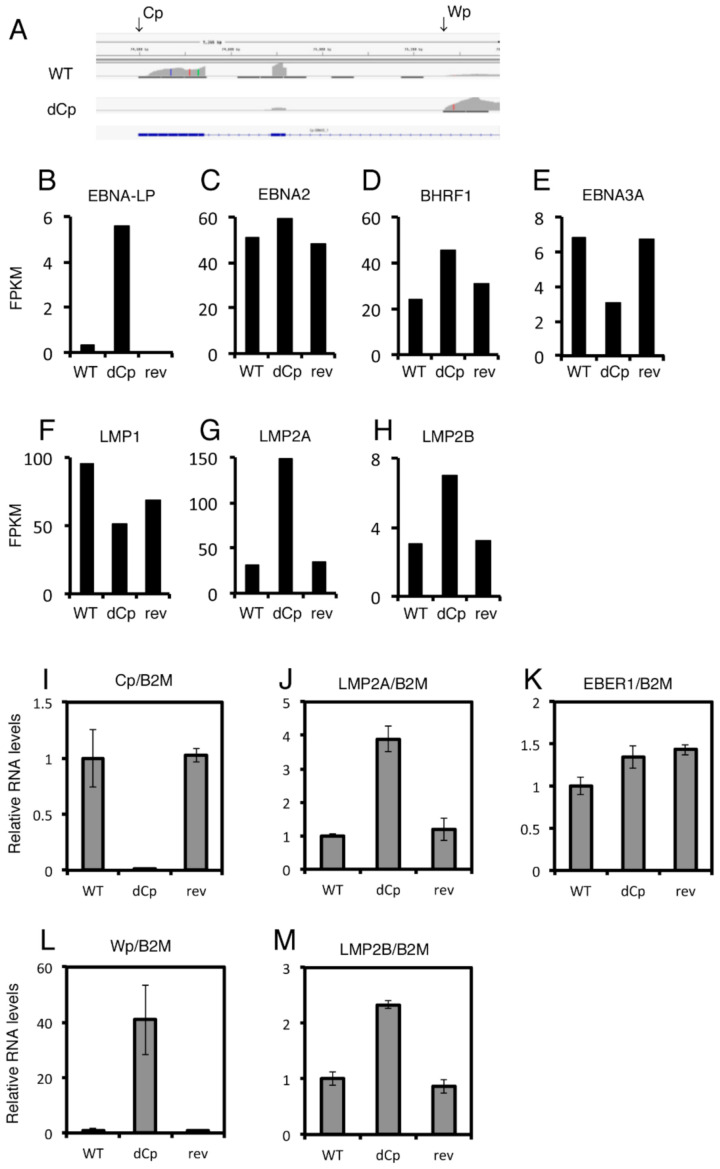
RNA sequencing (RNA-seq) and qRT–PCR of WT, dCp and rev virus-infected lymphoblastoid cell lines (LCLs). (**A**–**H**) RNA-seq analysis of infected LCLs. (**A**) read mapping of transcripts aligned to the Cp/Wp region of EBV. (**B**–**H**) fragments per kilobase of exon per million reads mapped (FPKM) data of EBV genes are depicted. (**I**–**M**) qRT–PCR of the LCLs. Relative RNA levels were standardized by β2-microglobulin (B2M). The mean and standard deviation values of three independent experiments are shown.

To gain insight into the mechanism underlying how Cp deletion enhanced B cell growth transformation, we next investigated the gene expression profile of LCLs infected with the WT, dCp and rev viruses using RNA sequencing (RNA-seq). The RNA-seq analysis clearly showed that LCLs infected with the dCp virus utilized the Wp instead of Cp for transcription of EBNAs (Figure 5A), as reported [16]. Figure 5B–H summarizes fragments per kilobase of exon per million reads mapped (FPKM) values of the LCLs, which reflect expression levels of mRNAs. Transcript of EBNA-LP was markedly induced in the dCp (Figure 5B), which is convincing because the dCp virus utilizes the Wp instead of Cp, and the exons of EBNA-LP are mapped to the BamHI-W region. In addition, EBNA-LP of B95-8 strain EBV is not functional due to a stop codon insertion [17], indicating that induction in EBNA-LP level does not account for the increased transformation by Cp deletion. Levels of the EBNA2 gene were comparable, possibly because the activity of the Wp in the dCp virus was high enough to recover the loss of the Cp. Expression of BHRF1 was slightly higher in the dCp LCLs (Figure 5D), while those of EBNA3A and LMP1 were lower due to unknown reasons (Figure 5D–F). Interestingly, we found that two LMP2 genes, namely the LMP2A, were notably induced (Figure 5G,H).

We then carried out a similar experiment, using primary B cells from a different donor, and analyzed the expression levels of EBV genes by qRT–PCR assays (Figure 5J–M). Figure 5I,L clearly showed that the Cp activity was lost, but instead, the Wp was induced in LCLs made by the dCp virus infection. Although the expression of the EBV noncoding RNA EBER1 was almost identical among the three viral strains (Figure 5K), dCp virus-infected LCLs showed higher LMP2A and LMP2B expression compared to controls (Figure 5J,M).

### 2.5. LMP2A Does Not Account for the Enhanced Immortalization Efficiency in the dCp

**Figure 6:**
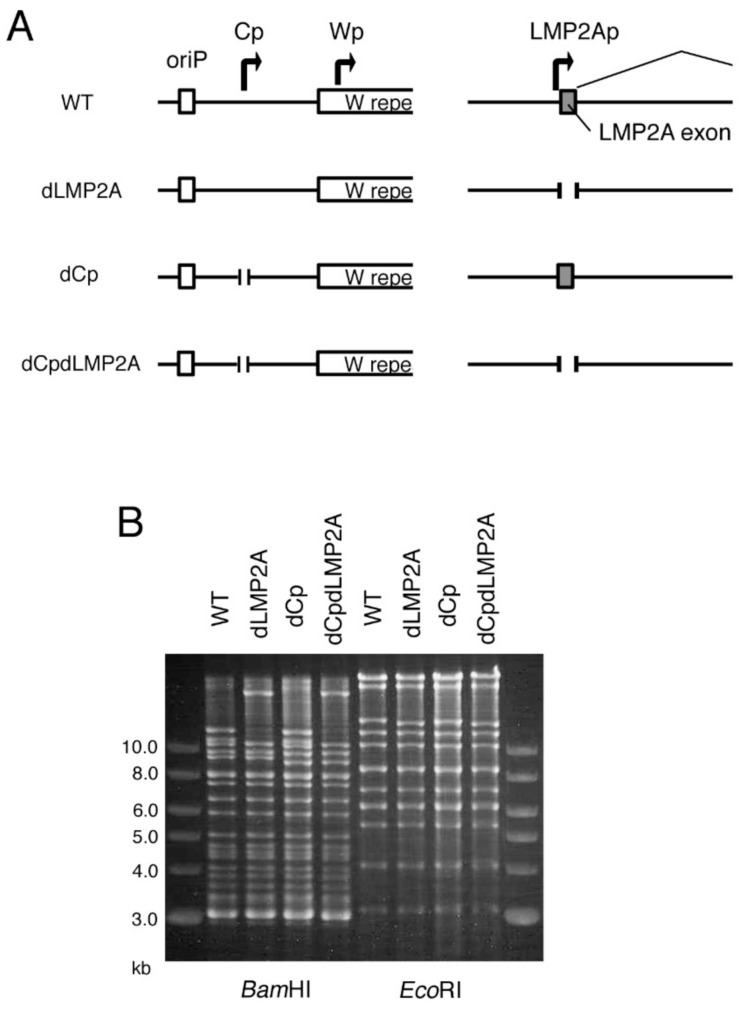
Construction of the latent membrane protein 2A-deficient (dLMP2A) and Cp/LMP2A double knockout (dCpdLMP2A) EBV mutants using the EBV-BAC system. (**A**) Schematic diagram of the EBV-BAC recombinant viruses. The first exons of the LMP2A gene of WT and dCp EBV constructs were removed to prepare dLMP2A and dCpdLMP2A, respectively. (**B**) Recombinant EBV-BAC DNA was digested by BamHI and EcoRI, and the products were examined by agarose gel electrophoresis.

LMP2A and 2B are not necessary for B cell immortalization [18], but LMP2A is important for efficient immortalization [19]. LMP2A mimics the BCR signaling pathway and provides pro-survival and anti-differentiation signals to B cells [20]. We speculated that Cp deletion might increase LMP2A expression, resulting in increased B cell growth transformation efficiency. To test this possibility, using the EBV-BAC system, we deleted the first exon of the LMP2A gene in WT and dCp viral genomes to make an LMP2A-deficient virus (dLMP2A) and LMP2A/Cp-deficient virus (dCpdLMP2A) (Figure 6A). The digestion pattern of the recombinant EBV-BAC DNAs demonstrates that the DNAs were prepared as intended, without any deletions (Figure 6B).

**Figure 7:**
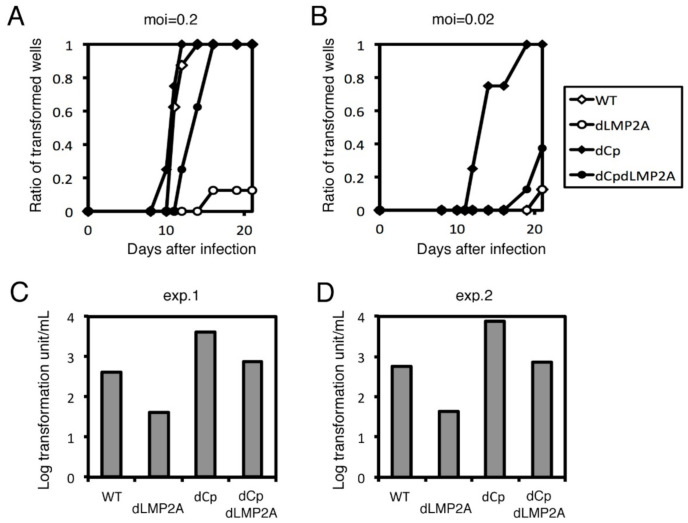
Transformation assay of dLMP2A and dCpdLMP2A viruses. Recombinant viruses (WT, dLMP2A, dCp, and dCpdLMP2A) were produced from HEK293 cells. After titer normalization of the viruses, 10-fold serial dilutions were prepared and used to separately infect PBMCs. For each dilution, 8 wells were prepared. (**A**,**B**) Ratio of transformed wells with a multiplicity of infection (MOI) of 0.2 (**A**) and 0.02 (**B**) over time. (**C**,**D**) Transformation efficiency at 3 weeks post-infection. Two independent experiments were carried out using PBMCs from two healthy donors (exp. 1 and exp. 2).

After transfection into HEK293 cells, recombinant viruses were prepared and titrated, followed by normalization of the titer, and infected to PBMCs (Figure 7). Deletion of Cp in the background of the WT virus caused faster clump formation (Figure 7B), as we already showed above (Figure 3); Cp deletion in the LMP2A knockout virus background also promoted faster transformation. The transformation efficiency of the double knockout (dCpdLMP2A) was similar to that of WT and lower compared to the dCp mutant. Single knockout of LMP2A (dLMP2A) resulted in lower transformation efficiency than WT virus (Figure 7C,D). Since the promotion of growth transformation via Cp deletion took place even in the absence of LMP2A, LMP2A was not necessary for the promotion of growth transformation via Cp deletion. Therefore, we concluded that the mechanism of enhanced B cell activation by Cp deletion does not involve LMP2A.

### 2.6. Viral BCL2 Homolog BHRF1 Does Not Account for the Enhanced Transformation Efficiency Seen in the dCp

**Figure 8:**
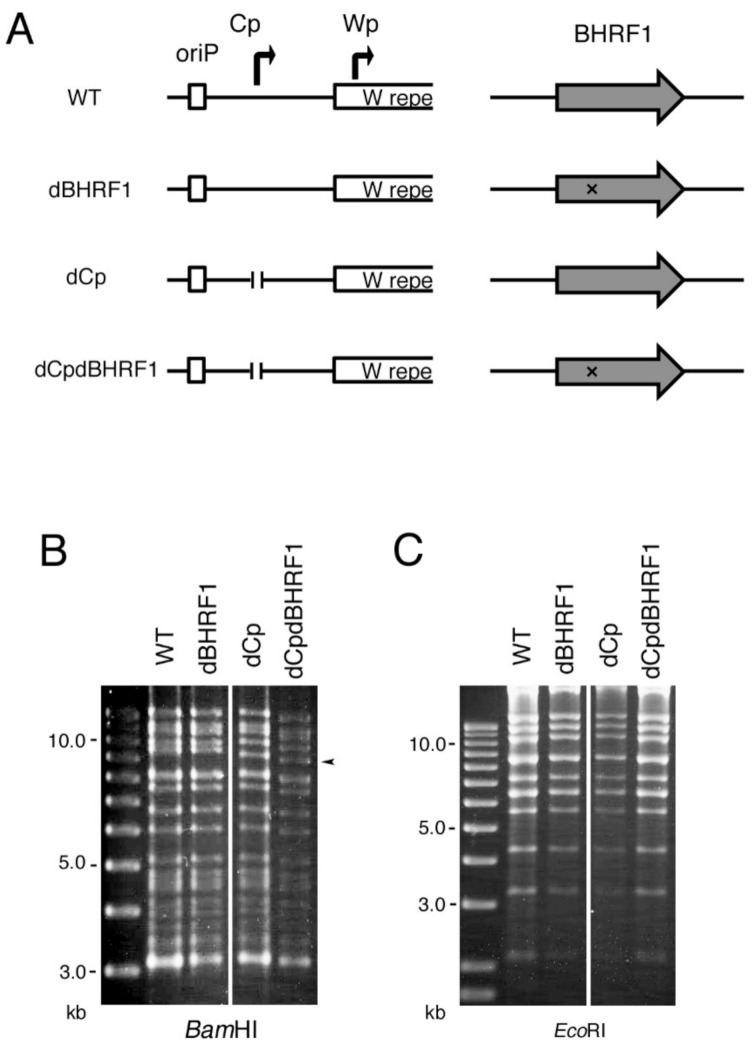
Construction of the BHRF1 deficient (dBHRF1) and Cp/BHRF1 double knockout (dCPdBHRF1) EBV mutants using the EBV-BAC system. (**A**) Schematic diagram of the EBV-BAC recombinant viruses. Two adenine residues were inserted between nucleotide 194 and 195 of BHRF1 amino acids to make a stop codon (T^194^AA). (**B**,**C**) Recombinant EBV-BAC DNA was digested by BamHI and EcoRI, and the products were examined by agarose gel electrophoresis.

BHRF1 is one of the EBV-coding BCL2 homologs. It functions as an anti-apoptotic protein by binding the pro-apoptotic proteins Bim, Puma and Bak [21,22,23]. BHRF1 is highly expressed by the Wp during primary infection [9] and enhances B cell growth transformation efficiency [24]. Wp-restricted BL cell line P3HR-1 requires BHRF1 for its survival and proliferation [25]. Because the dCp virus activates the Wp instead of Cp (Figure 5), and, indeed, the BHRF1 level was marginally higher in the dCp LCLs (Figure 5D), we speculated that BHRF1 might play a role in the enhanced transformation efficiency of the dCp virus. To test this, using the EBV-BAC system, a stop codon was inserted into the BHRF1 exon in the WT and dCp viral genomes to generate the BHRF1-deficient virus (dBHRF1) and BHRF1/Cp-deficient virus (dCpdBHRF1) (Figure 8A). Recombinant EBV-BAC DNA was digested by BamHI or EcoRI, and the digested products were confirmed by electrophoresis (Figure 8B,C).

**Figure 9:**
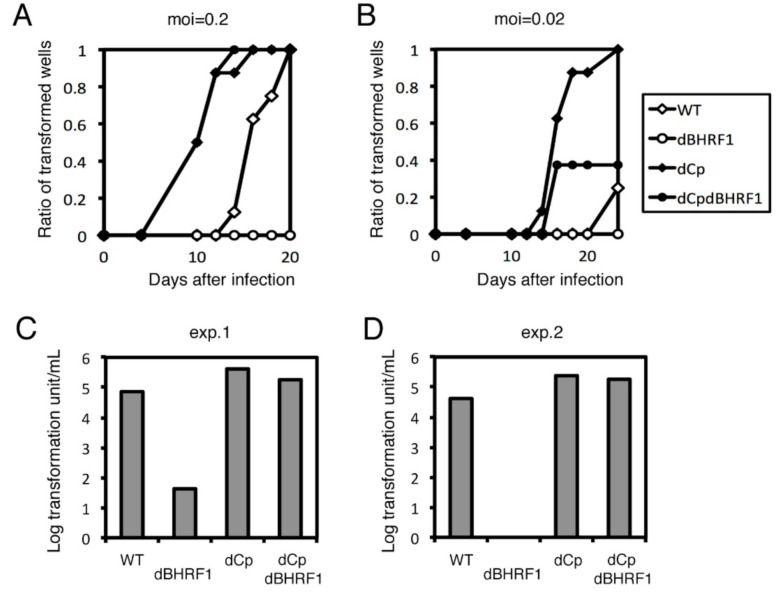
Transformation assay of dBHRF1 and dCpdBHRF1 viruses. Recombinant viruses (WT, dBHRF1, dCp, and dCpdBHRF1) were produced from HEK293 cells. After titer normalization of the viruses, 10-fold serial dilutions were prepared and used to separately infect PBMCs. For each dilution, 8 wells were prepared. (**A**,**B**) Ratio of transformed wells with a multiplicity of infection (MOI) of 0.2 (**A**) and 0.02 (**B**) over time. (**C**,**D**) Transformation efficiency at 3 weeks post-infection. Two independent experiments were carried out using PBMCs from two healthy donors (exp. 1 and exp. 2).

We conducted the B cell growth transformation assay again (Figure 9) in a similar manner, except that we concentrated virus stock by ultracentrifuge before infection because transformation by the BHRF1 mutant was very low. Deletion of Cp increased transformation efficiency compared to WT, and BHRF1 knockout (dCpdBHRF1) lowered the transformation efficiency to some extent (Figure 9A–D). However, transformation in dBHRF1 virus was severely impaired compared to WT virus (Figure 9A–D). In exp. 2, clump formation was completely absent in the BHRF1 knockout samples (Figure 9D). Therefore, these results indicate that the viral BCL2 homolog, BHRF1, was also unable to explain the enhanced transformation efficiency in the dCp virus because the promotion of growth transformation by Cp deletion took place even in the absence of the BHRF1 gene (Figure 9).

## 3. Discussion

Our results demonstrate that deletion of the EBV Cp enhances B cell growth transformation in vitro and results in faster expansion of EBV-associated lymphoproliferative disorders in vivo. There have only been a few reports of EBV genome mutations that promote the proliferation of EBV-infected cells. Our data imply that mutations of the viral genome can also contribute to EBV-associated tumor formation and proliferation.

Although deletions in the Cp region were associated with impaired transformation ability in previous studies [16], our dCp virus repeatedly demonstrated enhanced transformation efficiency. This may be due to differences in the deleted regions. The Cp deletion we used is very short and spans the transcription start site, making it likely that the region outside of the deletion contains a *cis*-acting sequence motif, which is crucial for transformation. The exact function of these regions needs to be clarified in further studies.

Transformation is generally mediated by enhancement of cellular growth and repression of cellular apoptosis. We focused on the contribution of the oncoprotein LMP2A and anti-apoptotic protein BHRF1 to B cell transformation, but neither could account for the enhanced transformation efficiency seen in the dCp virus. LMP2A gene was expressed abundantly in the dCp LCLs compared to WT (Figure 5). We still do not know why and how the LMP2 gene was overexpressed by deletion of the Cp, but the three-dimensional structure of the viral genome may play a role [26].

Disruption of BHRF1 had a very severe effect on B cell growth transformation compared to the mild effect seen in a previous report [24]. We speculate that this difference was attributable to the cells involved; the previous study used B cells derived from adenoids after T cell depletion, while we used PBMCs. The presence of germinal center B cells in adenoid tissue, and the depletion of T cells, may affect the severity of the response. Cells infected with the dBHRF1 virus showed severely impaired transformation efficiency compared to the WT virus, and the dCpdBHRF1 virus showed similar transformation efficiency to the dCp virus alone. This implies that, although BHRF1 does not play a role in the dCp-related higher transformation efficiency, the anti-apoptotic effect of BHRF1 could be compensated by Cp deletion. In this context, LMP2A was reported to induce degradation of anti-apoptotic proteins such as BHRF1, where BHRF1 targets protein Bim by activating ERK [27]. Elevated expression of LMP2A in the dCp virus-infected LCLs may affect this pathway. Additionally, the regulation of host genes by Cp deletion needs to be clarified in future studies.

In summary, we demonstrated that deletion of the Cp increased B cell growth transformation. This deletion was derived from a clinical sample and had the potential as a marker of malignancy. The role of Cp deletion in transformation efficiency suggests a novel tumorigenic mechanism, but further studies will be required to confirm this.

## 4. Materials and Methods

### 4.1. Cell Culture and Reagents

The cell lines HEK293, HEK293 EBV-BAC, and HEK293T were cultured in Dulbecco’s modified Eagle’s medium (Sigma-Aldrich, St. Louis, MO, USA) supplemented with 12% fetal bovine serum (FBS). Akata(−) cells were cultured in RPMI 1640 medium (Sigma-Aldrich) supplemented with 12% FBS. PBMCs were collected from healthy adult male donors who provided written informed consent, according to protocols approved by the Institutional Review Board of Nagoya University or Lonza Group AG. PBMCs and LCLs were maintained in RPMI 1640 medium (Sigma-Aldrich) supplemented with 12% FBS with 1% nonessential amino acids (Sigma-Aldrich).

### 4.2. Construction of the dCp, dLMP2A and dBHRF1 EBV-BAC Genome and Transfection into HEK293 Cells

The B95-8 EBV-BAC DNA sequence was a gift from W. Hammerschmidt [28]. To modify the EBV-BAC genome, homologous recombination was induced in *E.coli,* as described previously [29]. DNAs cassette containing neomycin-resistant and streptomycin-sensitive genes (Neo/St), with homology arms adjacent to the target sequences of Cp, LMP2A and BHRF1 were prepared by performing PCR using the rpsL-neo vector (Gene Bridges, Heidelberg, Germany) as template and primers, as follows: (Cp: forward 5′-CCTACGGGCGGGATTAATTACGCCTTGCTTACGCAAGCTCAGTTAATTCGCCCACGACTTGGCCTGGTGATGATGGCGGGATC-3′, reverse 5′-GCTGTTTCTTCAGTCCTAGAGGGAAGGAGAA TCACA TAAATTATTAATCTGCAAATAAAGTC AGAAGAACTCGTCAAGAAGG-3′, LMP2A: forward 5′-TGATCCTGTAGCGCCGCGGTTTCAGCATCACAGGTTATTTTGCCTGAAGCTTGCT GGGGCGTAGGCCTGGTGATGATGGCGGGATC-3′, reverse5′-GCCCTTATTATTGATGTGACTTG TGATGCAATAAATAAAAGTACAGATAGATGGCACTCTTACTCAGAAGAACTCGTCAAGAAGG-3′, BHRF1: forward 5′-CTGTAGTTCTGCGTTATCATGTGTTGCTTGAGGAGATAATTGAACGA AATTCAGAGACATGGCCTGGTGATGATGGCGGGATC-3′, reverse 5′-AGTTAAAATCCAGATCC ACATGTTCGGTGTGTGTTATAAATCTGTTCCAAGTTTCTGTAACAGAAGAACTCGTCAAGAAGG-3′). These DNA cassettes were introduced into *E. coli* containing EBV-BAC by electroporation. Homologous recombination was induced by Red/ET, and *E. coli* harboring rearranged mutant EBV-BAC was selected by kanamycin. Homologous recombination was performed again, replacing the Neo/St containing DNA cassette with the target sequence.

The target DNA for Cp deletion was made by overlapping PCR, using the following primers: (overlapping template oligos: 5′-CCTACGGGCGGGATTAATTACGCCTTGCTTACGCAAGCTCAGT TAATTCGCCCACGACTTCTTTATTTGC-3′, and 5′-GCTGTTTCTTCAGTCCTAGAGGGAAGGAG AATCACATAAATTATTAATCTGCAAATAAAGAAGTCGTGGG-3′, amplification primers 5′-CCT ACGGGCGGGATTAATTA-3′, reverse 5′-GCTGTTTCTTCAGTCCTAGA-3′). The target DNA for LMP2A deletion was also prepared by overlapping PCR, using the following primers (overlapping template oligos: 5′-TCCTGTAGCGCCGCGGTTTCAGCATCACAGGTTATTTTGCCTGAAGCTTGC TGGGGCGTAGTAAGAGTGC-3′, and 5′-GCCCTTATTATTGATGTGACTTGTGATGCAATAAAT AAAAGTACAGATAGATGGCACTCTTACTACGCCCCAG-3′, amplification primers 5′- TGATC CTGTAGCGCCGCGG-3′, reverse 5′- GCCCTTATTATTGATGTGACT-3′). The targeting vector for the BHRF1 sequence with a stop codon was made by inverse PCR using the following primers: follows (forward 5′-CTGTAGTTCTGCGTTATCATGTGTTGCTTGAGGAGATAATTGAACGAAATTCAGAGACATAATTACAGAAA-3′, reverse 5′-AGTTAAAATCCAGATCCACATGTTCGGTGTGTGTTATAAATC TGTTCCAAGTTTCTGTAATTATGTCTCTG-3′).

*E. coli* containing dCp, dLMP2A and dBHRF1 EBV-BAC was selected by streptomycin. The rev strain for the dCp virus was constructed by reinserting the Neo/St cassette and then replacing it with the WT Cp sequence. The recombination integrity was checked by PCR, sequence analysis, and analysis of band patterns in electrophoresis of the BamHI- or EcoRI-digested viral genome.

HEK293 cells were transfected with the recombinant EBV-BAC DNA using FuGENE^®^ HD transfection reagent (Promega, Madison, WI, USA). GFP-positive and hygromycin (150 µg/mL)-resistant colonies were picked up for further analyses.

### 4.3. Lytic Induction and Progeny Virus Titration

The BZLF1 expression plasmid was prepared as reported previously [30,31]. In brief, HEK293 EBV-BAC cells were transfected with BZLF1-expressing plasmid using FuGENE^®^ HD transfection reagent (Promega) to induce lytic cycle and progeny virus production. The supernatant containing progeny virus was concentrated by supercentrifuge and used to infect Akata(−) cells. After formalin fixation, the GFP-positive cell proportion was measured by FACS (Gallios, Beckman Coulter, Brea, CA, USA) for viral titration.

### 4.4. B Cell Growth Transformation Assay

Serial 10-fold dilutions of titer normalized virus solutions were used to infect 1.0 × 10^5^ PBMCs in a 96-well plate with cyclosporine A. After 3 weeks, the 50% transforming dose was determined by counting the number of wells showing LCL growth. PBMCs were obtained from LONZA and PRECISION FOR MEDICINE.

### 4.5. The EBV-Associated Lymphoproliferative Disorder Mouse Xenograft Model

Human cord blood mononuclear cells (1.0 × 10^7^) were infected with WT or dCp virus and immediately intraperitoneally injected into NOG mice (*n* = 6) [10]. On days 7, 14, 21 and 28, the body weight was measured and observed until these mice died because of EBV-associated lymphoproliferative disorder development. H-E staining was performed as described previously [10].

### 4.6. RNA Sequencing (RNAseq) and Quantitative Reverse Transcription-Polymerase Chain Reaction (qRT–PCR)

Total RNA was isolated from LCLs using RNeasy mini kit (Qiagen). For RNAseq, the quality of the RNA was checked by the tape station system (Agilent Technologies), followed by poly(A) RNA enrichment using poly(A) RNA magnetic isolation module (NEB). The sequencing library was prepared using NEBNext Ultra II RNA prep kit for Illumina (NEB) and sequenced using HiSeq X next-generation sequencer (Illumina). Data were processed and expressed as fragments per kilobase of exon per million reads mapped (FPKM) as described [10]. The qRT–PCR reactions were carried out using a one-step SYBR PrimeScript RT–PCR Kit II (TaKaRa) and real-time PCR system 7300 (Thermo Fisher Scientific), as described [32].

## 5. Conclusions

Deletion of the Cp, originally found in EBV-positive DLBCL, appeared to increase growth transformation efficiency. Although the molecular mechanism of this phenomenon is still elusive, loss of part of the EBV gene may increase tumorigenicity.

## Data Availability

Data are available upon request.
